# Biocomputational Prediction Approach Targeting FimH by Natural SGLT2 Inhibitors: A Possible Way to Overcome the Uropathogenic Effect of SGLT2 Inhibitor Drugs

**DOI:** 10.3390/molecules26030582

**Published:** 2021-01-22

**Authors:** Mutaib M. Mashraqi, Navaneet Chaturvedi, Qamre Alam, Saleh Alshamrani, Mosa M. Bahnass, Khurshid Ahmad, Amany I. Alqosaibi, Mashael M. Alnamshan, Syed Sayeed Ahmad, Mirza Masroor Ali Beg, Abha Mishra, Sibhghatulla Shaikh, Syed Mohd Danish Rizvi

**Affiliations:** 1 Department of Clinical Laboratory Sciences, College of Applied Medical Sciences, Najran University, Najran 61441, Saudi Arabia; mmmashraqi@nu.edu.sa (M.M.M.); saalshamrani@nu.edu.sa (S.A.); mmbohnass@nu.edu.sa (M.M.B.); 2 Biomolecular Engineering Laboratory, School of Biochemical Engineering, Indian Institute of Technology, Banaras Hindu University, Varanasi 221005, India; 14.navneet@gmail.com (N.C.); abham.bce@itbhu.ac.in (A.M.); 3 Department of Molecular and Cell Biology, Leicester Institute of Structural and Chemical Biology, University of Leicester Henry Wellcome Building, Lancaster Road Leicester, Leicester LE1 7HB, UK; 4 Medical Genomics Research Department, King Abdullah International Medical Research Center (KAIMRC), King Saud Bin Abdulaziz University for Health Sciences, King Abdulaziz Medical City, Ministry of National Guard Health Affairs, Riyadh 11426, Saudi Arabia; alamqa@ngha.med.sa; 5 Department of Animal Medicine (Infectious Diseases), Faculty of Veterinary Medicine, Zagazig University, Zagazig 44519, Egypt; 6 Department of Medical Biotechnology, Yeungnam University, Gyeongsan 38541, Korea; ahmadkhursheed2008@gmail.com (K.A.); sayeedahmad4@gmail.com (S.S.A.); mirzamasroor1986@gmail.com (M.M.A.B.); 7 Department of Biology, College of Science, Imam Abdulrahman bin Faisal University, P.O. Box 1982, Dammam 31441, Saudi Arabia; amgosaibi@iau.edu.sa (A.I.A.); malnamshan@iau.edu.sa (M.M.A.); 8 Department of Pharmaceutics, College of Pharmacy, University of Hail, P.O. Box 2440, Hail 81451, Saudi Arabia

**Keywords:** sodium–glucose co-transporters 2, FimH, uropathogenic bacteria, urinary tract infections, diabetes

## Abstract

The Food and Drug Administration (FDA) approved a new class of anti-diabetic medication (a sodium–glucose co-transporter 2 (SGLT2) inhibitor) in 2013. However, SGLT2 inhibitor drugs are under evaluation due to their associative side effects, such as urinary tract and genital infection, urinary discomfort, diabetic ketosis, and kidney problems. Even clinicians have difficulty in recommending it to diabetic patients due to the increased probability of urinary tract infection. In our study, we selected natural SGLT2 inhibitors, namely acerogenin B, formononetin, (−)-kurarinone, (+)-pteryxin, and quinidine, to explore their potential against an emerging uropathogenic bacterial therapeutic target, i.e., FimH. FimH plays a critical role in the colonization of uropathogenic bacteria on the urinary tract surface. Thus, FimH antagonists show promising effects against uropathogenic bacterial strains via their targeting of FimH’s adherence mechanism with less chance of resistance. The molecular docking results showed that, among natural SGLT2 inhibitors, formononetin, (+)-pteryxin, and quinidine have a strong interaction with FimH proteins, with binding energy (∆G) and inhibition constant (ki) values of −5.65 kcal/mol and 71.95 µM, −5.50 kcal/mol and 92.97 µM, and −5.70 kcal/mol and 66.40 µM, respectively. These interactions were better than those of the positive control heptyl α-d-mannopyranoside and far better than those of the SGLT2 inhibitor drug canagliflozin. Furthermore, a 50 ns molecular dynamics simulation was conducted to optimize the interaction, and the resulting complexes were found to be stable. Physicochemical property assessments predicted little toxicity and good drug-likeness properties for these three compounds. Therefore, formononetin, (+)-pteryxin, and quinidine can be proposed as promising SGLT2 inhibitors drugs, with add-on FimH inhibition potential that might reduce the probability of uropathogenic side effects.

## 1. Introduction

Globally, diabetes mellitus is one of the most prevalent metabolic diseases and is estimated to increase to 552 million cases by 2030 [1]. Diabetes has been considered to enhance vulnerability to infectious diseases and has also been linked with an enhanced risk of death from infectious illness in some [2,3], but not all [4], investigations.

Sodium–glucose cotransporter-2 (SGLT2) inhibitors are a novel group of drugs used to treat patients with type 2 diabetes mellitus (T2DM). These drugs exert their effects by preventing glucose reabsorption at the proximal renal tubule and enhancing the excretion of urinary glucose [5]. Owing to the elevated urine glucose levels, SGLT2 inhibitors enhance the risk of urinary tract infections (UTIs) [6]. In addition, pharmacologically-induced urine glucose levels with SGLT2 inhibitors in diabetic patients might further sustain bacterial growth [7]. By themselves, SGLT2 inhibitors can possibly enhance the risk of UTIs and susceptibility to genital infections when used to manage patients [8]. SGLT2 inhibitors might cause serious UTIs, as the FDA warned in December 2015 [9]. Empagliflozin and canagliflozin are the preferred drugs suggested for T2DM patients with established cardiovascular disease [10,11]. The United States-based public safety advisory reported 19 cases of fatal renal or blood infection from March 2013 to October 2014. The origin of these cases was traced to a UTI induced by SGLT2 inhibitor intake [9].

Bacterial pili are proteinaceous projections extending from the bacterial cell surface and are used for attachment and cell motility [12]. Gram-negative bacteria use Type 1 pili to adhere to the host tissue and, therefore, Type 1 pili have been established as an important virulence factor in UTIs. Type 1 pili are made up of repeated subunits of FimA protein. These subunits are arranged to form a helical wound cylinder that composes a thick pilus rod. The distal flexible tip of the pilus rod is comprised of a single copy of proteins—FimF and FimG—while the tip adhesin bears FimH. The distinct binding of FimH (terminal adhesin) to mannosylated host glycoproteins mediates the adhesion of bacterial pathogens to the host tissue. UTI pathogenesis is caused by the FimH and, therefore, is a promising curative target [13].

Acerogenin B is a cyclic diarylheptanoid obtained from the bark of *Acer nikoense*, and it has been found to inhibit both SGLT1 and SGLT2 [14]. Kurarinone and formononetin are flavonoids isolated from the dried root of *Sophora flavescens.* Kurarinone has demonstrated inhibitory activity against both SGLT1 and SGLT2; however, formononetin was reported to inhibit only SGLT2 and not SGLT1 [15]. Quinidine is isolated from the bark of the cinchona tree and (+)-pteryxin is extracted from the plant *Peucedanum* spp. Recently, both of these natural compounds have been found to inhibit both SGLT1 and SGLT2 [16]. Heptyl α-d-mannopyranoside is a FimH antagonist [17] and, in this study, it was used as a reference compound. 

In the present study, we explored natural SGLT2 inhibitors (acerogenin B, kurarinone, formononetin, quinidine, and (+)-pteryxin) that might have less severe uropathogenic side effects than does the approved SGLT2 inhibitor canagliflozin, also known as gliflozins. We speculated that formononetin, (+)-pteryxin, and quinidine would be promising SGLT2 inhibitors with less severe uropathogenic side effects.

## 2. Methodology

### 2.1. SGLT2 Inhibitors and Target Protein Structure Retrieval

The 3-dimensional structure of FimH was taken from the protein data bank (PDB ID: 4AV5), while the 3-dimensional structure of SGLT2 was made by employing the SWISS-MODEL Workspace after retrieving its amino acid sequence from Uniprot (P31639). The PDB structure 2XQ2 was used as a template and the generated model was validated using various in silico tools, viz., SAVES v6.0 and VADAR (Volume, Area, Dihedral Angle Reporter). The PDB structure of the compounds canagliflozin (CID: 24812758), acerogenin B (CID: 10913542), formononetin (CID: 5280378), (−)-kurarinone (CID: 10812923), (+)-pteryxin (CID: 511787), quinidine (CID: 441074), and the FimH antagonist heptyl α-d-mannopyranoside (CID: 11300413) were retrieved from the PubChem database.

### 2.2. Physicochemical Properties and Toxicity Potential Prediction

The physicochemical properties and toxicity potential of SGLT2 inhibitors and FimH antagonists were calculated by applying the Orisis Datawarrior property explorer tool (http://www.openmolecules.org/datawarrior/download.html). At first, molecular weight, the number of rotatable bonds, the number of hydrogen bond acceptors and donors, cLogP value, topological polar surface area (TPSA), and the Lipinski’s rule violation [18] were estimated to check physicochemical parameters. Later, the method outlined by Zhao et al. [19] was applied to calculate percentage of absorption; here, the following formula was used: absorption % = 109 − (0.345 × TPSA). Toxicity was also predicted for SGLT2 inhibitors and FimH antagonists by using toxicity features such as irritability, reproductive effects, tumorigenicity, and mutagenicity. Orisis Datawarrior property explorer tool toxicity predictions are based on comparative analysis of our tested compounds with the pre-estimated set of known structural molecules.

### 2.3. Molecular Docking

The ligands were docked to the SGLT2 and FimH proteins using “AutoDock 4.2” by following the protocol described by Rizvi et al. [20]. To minimize the energy usage of the ligand molecules, a Merck molecular force field (MMFF94) was employed. The ligand atoms were added with Gasteiger partial charges. Docking calculations were done on the target proteins. Essential hydrogen atoms, Kollman united atom type charges and solvation parameters were added by using AutoDock tools. Consequently, the binding pocket was added with conserved water molecules to mimic the *in vivo* environment. An auto grid program was used to generate the affinity (grid) maps sized at 60 × 60 × 60°, the aim of which was to target the grid coordinates in the catalytic site of the target protein (SGLT2 and FimH). The x, y, and z coordinate values for the FimH protein targeting the catalytic site were taken as 3.305, −16.68, and −13.57, respectively. Docking simulations were done using the Lamarckian genetic algorithm and the Solis and Wets local search method. The initial position, orientation, and torsions of the ligands were set randomly. Each docking experiment was derived from 100 different runs that were set to terminate after a maximum of 2,500,000 energy evaluations. The population size was set to 150. The Discovery Studio Visualizer 2.5 (Accelrys, San Diego, CA, USA) was used to generate the final figures. 

### 2.4. LIGPLOT+ ANALYSIS

After the completion of all docking experiments, the best combination of ligand–FimH and ligand–SGLT2 was selected and analyzed using LIGPLOT+ Version v.2.1 (EMBL-EBI, Cambridgeshire, UK). The hydrogen and hydrophobic interactions between the important amino acid residues of FimH/SGLT2 with each ligand were analyzed by LIGPLOT. For each interaction, the 3-D structures generated were converted into 2-D figures by applying the LIGPLOT algorithm.

### 2.5. Molecular Dynamics Simulation

System building: GROMACS 4.6.7 packages [21,22] were used to prepare the system and perform molecular dynamics (MD) simulations using the gromos53a6 force field [23]. The protein solute was solvated by explicit SPC216 water [24] in a dodecahedral box with a margin of 10 Å between the solute and the box walls. Systems were brought to neutrality by the addition of sodium counter ions.

Simulation detail: A 10 Å cut-off distance was taken under the particle-mesh Ewald method [25] to calculate long-range electrostatic interactions, and a 10 Å cut-off distance was also considered to evaluate van der Waals interactions. The LINCS algorithm of fourth-order expansion was used to constrain bond lengths [26]. The steepest descent algorithm was applied to optimize the removal of steric clashes between atoms for 10,000 steps. The system was equilibrated for 1 ns with position restraints on all heavy atoms. Berendsen weak coupling schemes were used to maintain the system at 300 K and 1 atom, using separate baths for the system. Initial velocities were generated randomly using a Maxwell–Boltzmann distribution corresponding to 300 K. Finally, the production run was performed for 20 ns. Furthermore, xmgrace (http://plasma-gate.weizmann.ac.il) was used for preparing graphs. Ligand topology preparation was implemented by using the PRODRG server, using the option specifying no chirality, full charge, and no energy minimization [27].

Trajectory analysis: The g_rms tool of the GROMACS package was used to calculate the root-mean-square deviation (RMSD) of each trajectory. The covariance matrix and eigenvectors of the trajectories were calculated through g_covar and g_anaeig programs. 

## 3. Results and Discussion

### 3.1. Physicochemical Properties and Molecular Docking

Many varieties of plant-based natural compounds have been reported, which have significant anti-diabetic effects. In streptozotocin-stimulated diabetic mice, bakuchiol (a polyphenol compound) decreases glucose levels and enhances serum insulin levels [28]. Caffeine (an alkaloid) lowers glucose, induces insulin secretion in vitro, and improves glucose absorption in skeletal muscle [29]. Vanillic acid isolated from *Fagara tessmannii Engl.* (Family: Rutaceae) exhibits α-glucosidase inhibitory actions in vitro [30]. Christinin-A is a triterpenoidal saponin glycoside that exhibits an anti-hyperglycemic effect in both type 1 and type 2 diabetic rats [31].

Molecular docking has an important role in drug discovery, assisting in digging out the active or lead compounds from a library of natural compounds [32]. It is one of the most widely used virtual screening tools, particularly when the three-dimensional structure of the target protein is available. Docking enables the prediction of both ligand–target binding affinity and the structure of the protein–ligand complex, which are useful for optimizing the lead [33,34].

Prior to the molecular docking study, we checked the physicochemical properties and toxicity potential of SGLT2 inhibitors and FimH antagonists (Table 1 and Table 2). The selected SGLT2 inhibitors were known drug canagliflozin and natural SGLT2 inhibitors, namely, acerogenin B, formononetin, (−)-kurarinone, (+)-pteryxin, and quinidine, while heptyl α-d-mannopyranoside was selected as an FimH antagonist. During physicochemical property assessment, we found that out of all the compounds tested, (−)-kurarinone showed one violation of the Lipinski rule [18], i.e., a cLogP value (Logarithm of compound partition coefficient between n-octanol) higher than 5 (Table 1). On the other hand, all the tested compounds showed no toxicity except (+)-pteryxin. (+)-pteryxin was predicted to have a high irritant effect with no mutagenic, tumorigenic, or reproductive toxicity (Table 2). 

**Figure 1:**
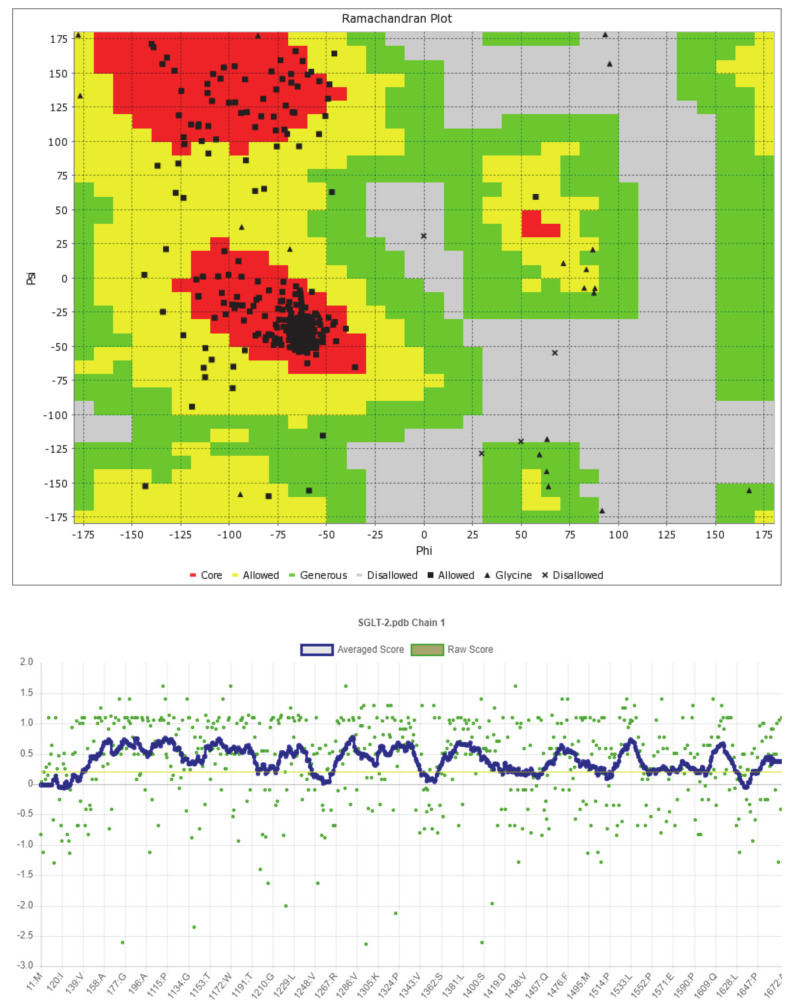

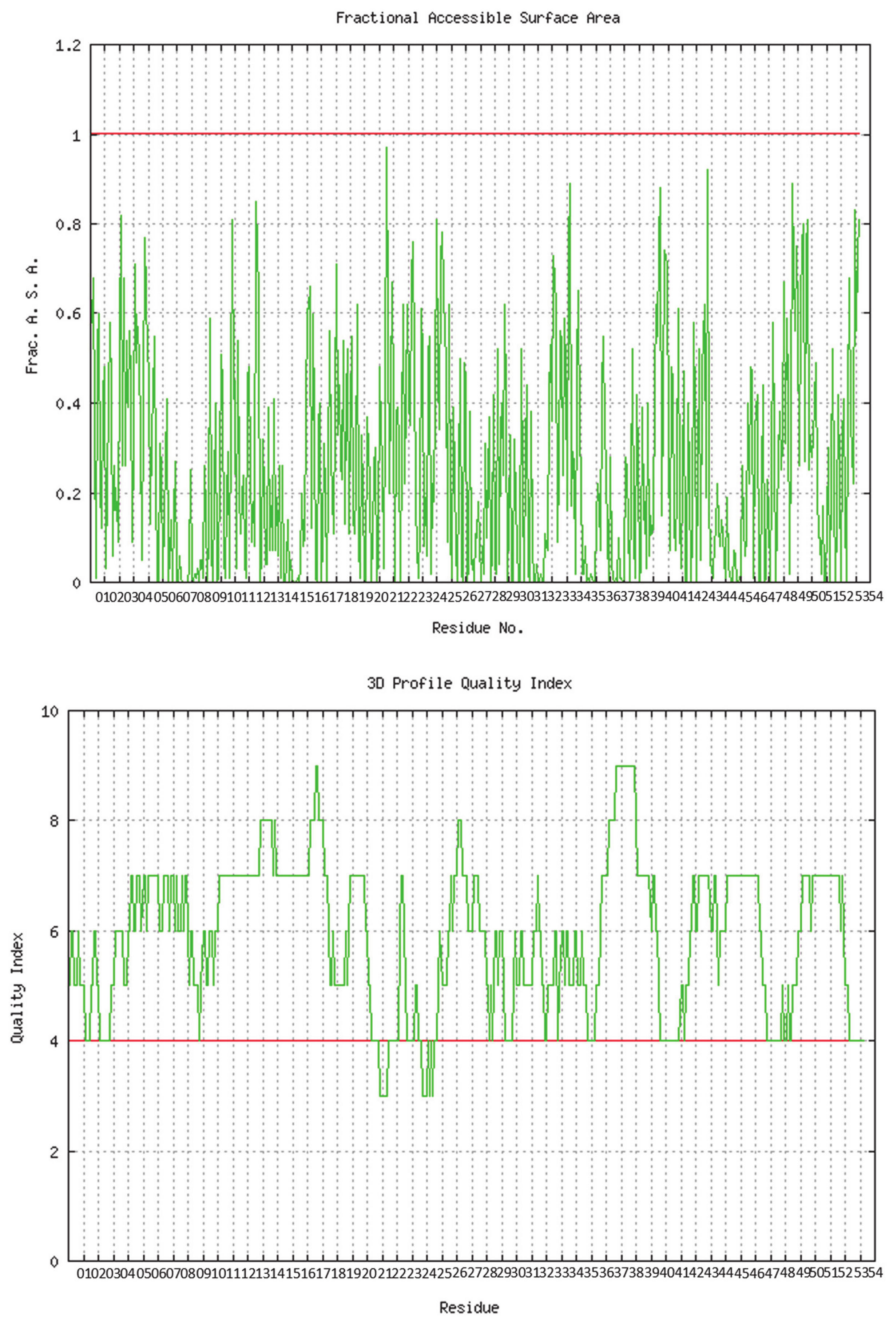

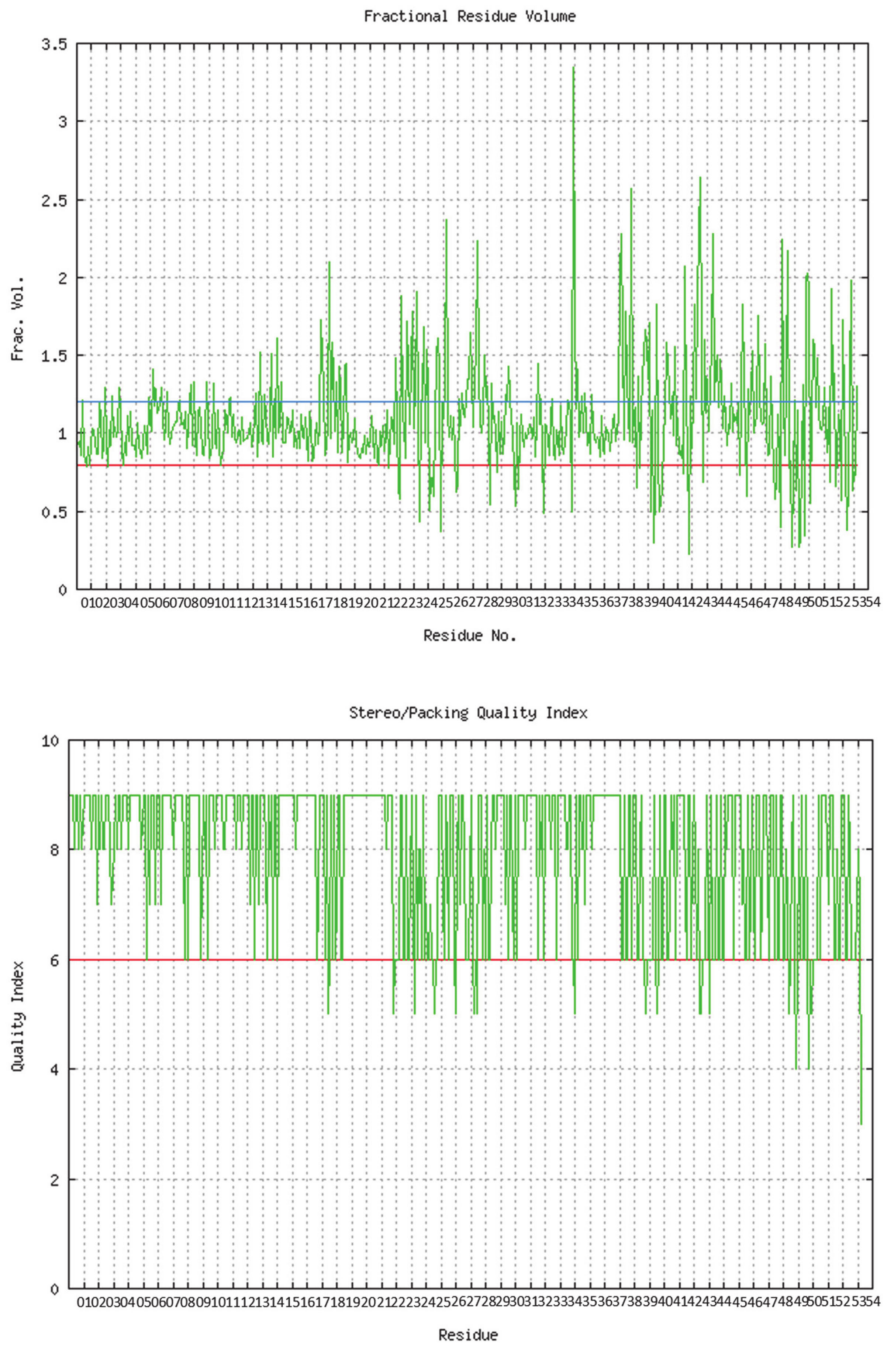
Validation of the predicted 3-D structure of SGLT2.

The predicted model of SGLT2 revealed that 81.25 percent of the residues had an average 3D-1D score of ≥0.2, resulting in a “pass” in SAVES v6.0.0 by the VERIFY3D tool. In addition, the Ramachandran plot (showing 93% of the residues in the allowed region), fractional accessible surface area, stereo/packing quality index, fractional residue volume, and 3-D profile quality index (produced by the VADAR 1.8 server) showed that the predicted 3-D model was within an appropriate range (Figure 1).

**Figure 2:**
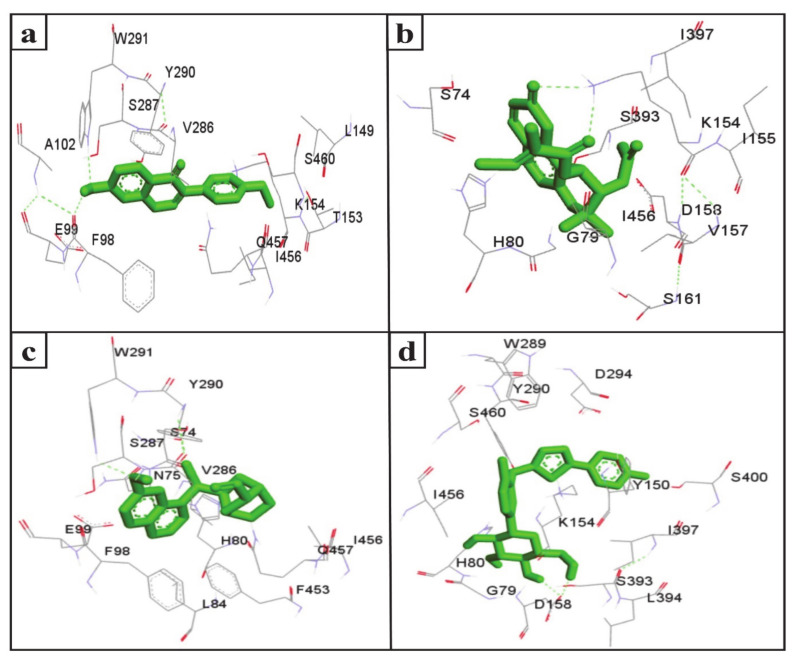
The amino acid residue of SGLT2 interacting with formononetin (**a**), (+)-pteryxin (**b**), quinidine (**c**), and canagliflozin (**d**). The ligands (formononetin, (+)-pteryxin, quinidine, and canagliflozin) are represented as green stick forms and hydrogen bonds are indicated as green dashed lines.

**Figure 3:**
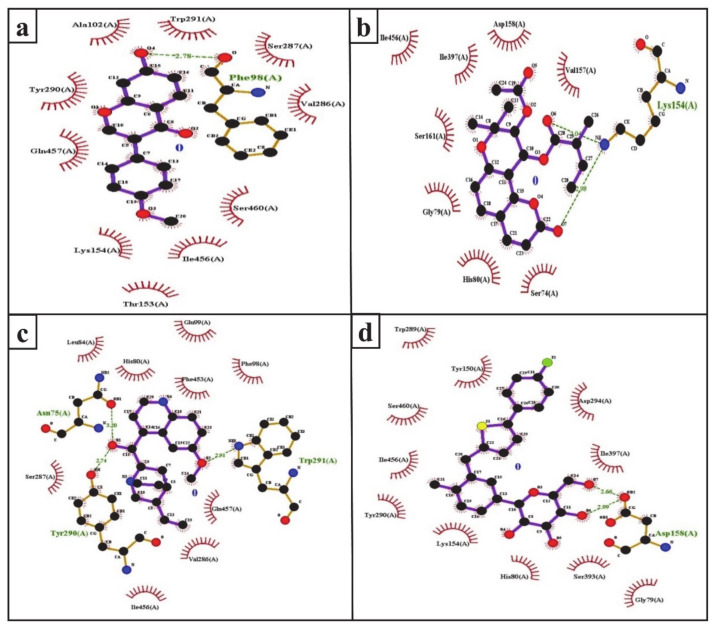
Ligplot analysis of formononetin (**a**), (+)-pteryxin (**b**), quinidine (**c**), and canagliflozin (**d**) in terms of their interaction with SGLT2. The amino acid residues forming hydrophobic interactions are shown as red arcs while the hydrogen bonds are shown as green dashed lines with indicated bond lengths.

The inhibition of SGLT2 has been considered a novel pharmacotherapy for T2DM treatment [35]. Accordingly, molecular docking studies have revealed that the natural SGLT2 inhibitors formononetin, (+)-pteryxin, and quinidine were efficiently bound with SGLT2. Formononetin was found to interact with the F98, E99, A102, L149, K152, T153, V286, S287, Y290, W291, I456, Q457, and S460 binding pocket residues of SGLT2 (Figure 2a); while the S74, G79, H80, K154, I155, V157, D158, S161, S393, I397, and I456 residues of SGLT2 were observed to bind with (+)-pteryxin (Figure 2b). Furthermore, quinidine was found to interact with the S74, N75, H80, L84, F98, E99, V286, S287, Y290, W291, F453, I456, and Q457 residues of SGLT2 (Figure 2c). Consistent with this, the amino acid residues H80, F98, T153, K154, V157, D158, V290, I397, F453, I456, Q457, and S460 were shown to be important for the inhibition of SGLT2 [36]. Amino acid residues H80, V286, Y290, W291, and I456 are the main hydrophobic residues of SGLT2, interacting with the dock inhibitors formononetin, (+)-pteryxin and quinidine (Figure 3a–c). This concurs with a previous report wherein the amino acid residues H80, Y290, and I456 of SGLT2 have also been reported have a hydrophobic interaction with the inhibitor [36,37]. The binding energy (BE) for the catalytic domain interactions of formononetin–SGLT2, (+)-pteryxin–SGLT2, and quinidine–SGLT2 were found to be −7.63 kcal/mol, −9.01 kcal/mol, and −8.77 kcal/mol, respectively, while their inhibition constants (Ki) were 2.57 µM, 0.245 µM, and 0.371 µM, respectively (Table 3).

The FDA approved canagliflozin, an SGLT2 inhibitor, for use in T2DM treatment in 2013 [38]. In the present study, canagliflozin was used as a positive control against SGLT2. Canagliflozin was observed to bound with G79, H80, Y150, K154, D158, W289, Y290, D294, S393, I397, S460, and I456 residues of SGLT2 (Figure 2d and Figure 3d). Interestingly, these amino acids of SGLT2 have also been found to interact with SGLT2 inhibitors (formononetin, (+)-pteryxin, and quinidine). I456 was one of the most reactive common hydrophobic residues of SGLT2, interacting with formononetin, (+)-pteryxin, and quinidine, as well as canagliflozin (Figure 3a–d). The Y290 residue of SGLT2 was found to make H-bonds with quinidine, while the same residue was observed to hydrophobically interact with formononetin and canagliflozin (Figure 3a,c,d). In addition, G79, H80, and I397 were the common hydrophobic interacting residues of SGLT2 with (+)-pteryxin and canagliflozin (Figure 3b,d).

The function of human SGLT1 protein is dramatically affected by amino acid at position 457. It has been shown that residue 457 (i.e., Q457) in human SGLT1 directly interacts with sugar for its reabsorption [39,40]. The amino acid sequences of SGLT1 and SGLT2 revealed that both of these proteins have glutamine at the residue 457 position, and glucose–galactose malabsorption occurs due to mutation in the glutamine residue (Q457) [41]. Consistent with this, in the present study, formononetin and quinidine were observed to interact with the Q457 residue that is suggested to impair SGLT2 function.

Since the discovery of the first natural SGLT2 inhibitor (i.e., phlorizin), several other synthetic glucoside analogs have been developed [42]. Tofogliflozin is a selective SGLT2 inhibitor that enhances urinary glucose excretion in a dose-dependent manner [43]. Luseogliflozin is an orally active second-generation SGLT2 inhibitor that has protective effects on pancreatic beta-cell mass and function [44]. Furthermore, efforts have been made to find new active natural ingredients from *S. Flavescents* [15], alkaloids from *A. macrophylla* [45], and *Schisandrae Chinensis Fructus* for the development of SGLT2 inhibition [46]. A 4-O-methyl derivative of sergliflozin-A has been reported to exhibit SGLT2 inhibition activity [47].

SGLT2 inhibitors are some of the most promising anti-diabetic agents introduced into clinical practice over the last decade. The therapeutic benefits of SGLT2 inhibitors include weight loss, a reduction in blood pressure, and an enhancement in high-density lipoprotein level [48,49]. However, the SGLT2 inhibitors dapagliflozin, canagliflozin, and empagliflozin have been found to cause UTIs and genital infections in diabetic patients [50,51,52]. Additionally, the FDA has issued warnings about the possible UTI-inducing side effects of SGLT2 inhibitors in December 2015 [9].

**Figure 4:**
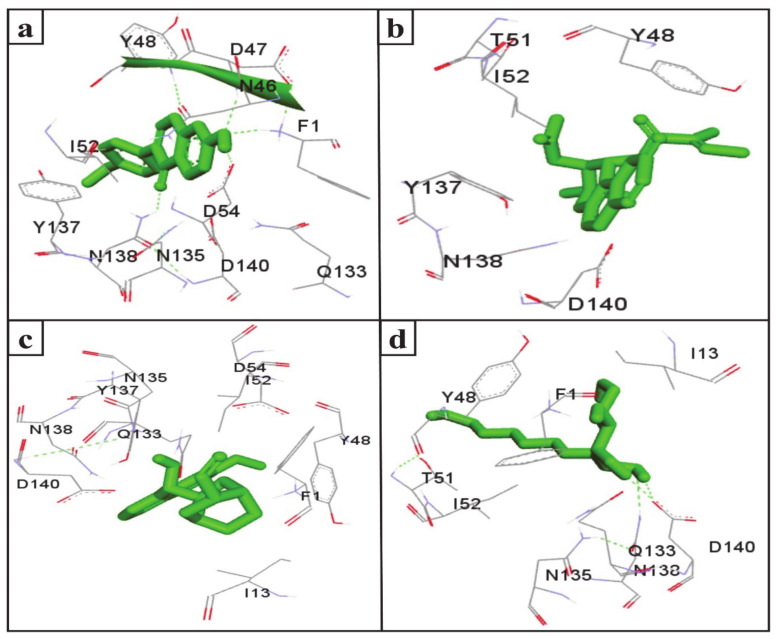
The amino acid residue of FimH’s interaction with formononetin (**a**), (+)-pteryxin (**b**), quinidine (**c**), and heptyl α-d-mannopyranoside (**d**). The ligands (formononetin, (+)-pteryxin, quinidine, and heptyl α-d-mannopyranoside) are represented as green stick forms and hydrogen bonds are represented as green dashed lines.

UTIs pose a severe medical issue worldwide, with more than 85% of UTIs caused by uropathogenic *Escherichia coli* [53]. FimH is a bacterial adhesin that facilitates the colonization of uropathogenic *E. coli* on the cell surface of the human and murine bladder and leads to the formation of biofilm [54]. Therefore, this adhesin has been considered a virulence factor and a promising therapeutic target for the treatment of UTIs [55]. Interestingly, the docking results indicate that the SGLT2 inhibitors formononetin, (+)-pteryxin, and quinidine show strong binding with FimH. FimH was found to interact with formononetin through 11 amino acid residues, namely F1, N46, D47, Y48, I52, D54, Q133, N135, Y137, N138, and D140 (Figure 4a), while (+)-pteryxin was found to interact with 6 amino acid residues, namely Y48, T51, I52, Y137, N138, and D140 (Figure 4b). Similarly, 10 amino acid residues, namely the F1, I13, Y48, I52, D54, Q133, N135, Y137, N138, and D140 residues of FimH, were found to interact with quinidine (Figure 4c). The BE for formononetin–FimH, (+)-pteryxin–FimH, and quinidine–FimH interactions were found to be −5.65 kcal/mol, −5.50 kcal/mol, and −5.70 kcal/mol, respectively. The corresponding estimated inhibition constants (Ki) for the above-mentioned complexes were determined to be 71.95 µM, 92.97 µM, and 66.40 µM, respectively (Table 3).

**Figure 5:**
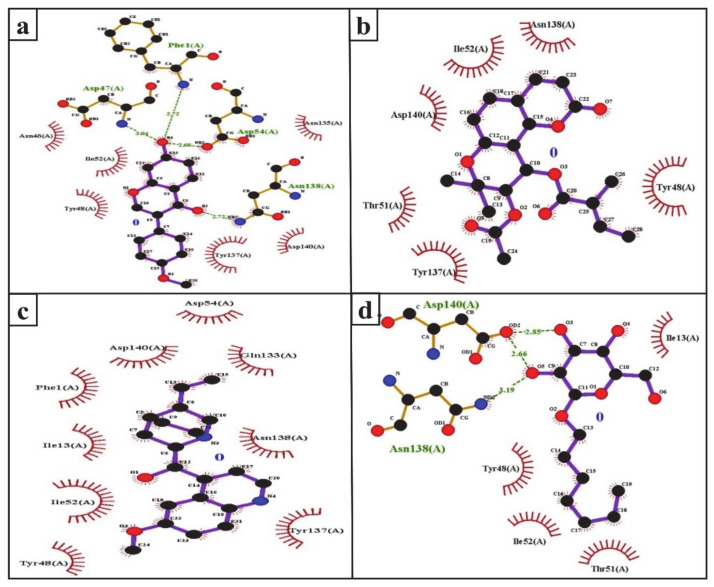
Ligplot analysis of the interactions of formononetin (**a**), (+)-pteryxin (**b**), quinidine (**c**), and heptyl α-d-mannopyranoside (**d**) with SGLT2. The amino acid residues forming hydrophobic interactions are shown as red arcs while the hydrogen bonds are shown as green dashed lines with indicated bond lengths.

The FimH protein has two domains, the C-terminal pilin domain and the N-terminal lectin domain. The FimH lectin domain has a mannose-binding pocket (N46, D47, D54, Q133, N135, N138, and D140), in which sugar contributes to the formation of various hydrogen bonds with FimH. Hydrophobic regions are present in the surrounding region of the mannose-binding pocket and consist of hydrophobic support (F1, I13, and F142), the tyrosine gate (Y48, I52, and Y137), and the residue T51 [56]. Consistent with this, in the present study, the D47, D54, and N138 of the FimH protein were involved in hydrogen binding with formononetin (Figure 5a), while Y48, I52, Y137, and D140 were the common hydrophobic amino acid residues interacting with formononetin, (+)-pteryxin and quinidine (Figure 5a–c).

In the present study, heptyl α-d-mannopyranoside (a FimH antagonist) was used as a positive control against the FimH protein. Molecular docking analysis revealed that amino acid residues, namely the F1, I13, Y48, T51, Q133, N135, N138, D140, and I52 residues of the FimH protein, have a vital role in binding with heptyl α-d-mannopyranoside (Figure 4d and Figure 5d). The main FimH binding pocket amino acid residues are F1, N46, D47, D54, E133, N135, D140, and F142, and mutation in these individual main residues leads to them affecting FimH function and reducing its virulence [57,58]. Interestingly, we found that these amino acid residues of FimH were also determined to interact with natural SGLT2 inhibitors (formononetin, (+)-pteryxin, and quinidine).

O- and C-linked alpha-d-mannosides with hydrophobic and aryl substituents are effective FimH antagonists. The substitution of biphenyl derivatives may provide additional advantages for the FimH antagonist molecule. The addition of 1,10-biphenyl pharmacophore and various aglycone atoms enhanced the alpha-d-mannose derivatives’ suitability as FimH inhibitors [59,60]. Further, glycomimetics based on mannose scaffolding has also been synthesized and widely studied for their aptitude as FimH antagonists [61,62]. Besides synthetic compounds, natural substances, like cranberry and its derivatives (such as myricetin, proanthocyanidin (PAC)-standardized cranberry extract, and polyphenol metabolites extracted from PAC), have anti-adhesive effects on uropathogenic *E. coli* [63].

In docking experiments, it is usually crucial to look for a ligand that can bind efficiently with the receptor, using Gibbs free energy as a parameter of better binding [64,65]. The strength of an interaction between a ligand and a receptor is measured in terms of the free energy of binding. The lowest BE is the output of the efficient binding of the drug/ligand to the active site of the receptor [66]. A higher (negative) BE is a sign of efficient interaction between the ligand and the receptor [67]. Accordingly, in the present study, formononetin, (+)-pteryxin, and quinidine exhibited strong interaction with the FimH protein, with a high BE relative to that of the positive control heptyl α-d-mannopyranoside, suggesting that these compounds could be promising SGLT2 inhibitors with less severe uropathogenic side effects. 

Diabetic ketoacidosis and its associated events have been reported at a low frequency in T2DM patients treated with canagliflozin [68]. Interestingly, in the present study, the BE of canagliflozin with FimH relative to the other SGLT2 inhibitor indicates that canagliflozin has less potency in inhibiting the FimH protein, thereby having a high susceptibility to causing diabetic ketosis and UTIs.

### 3.2. Root Mean Square Deviation

**Figure 6:**
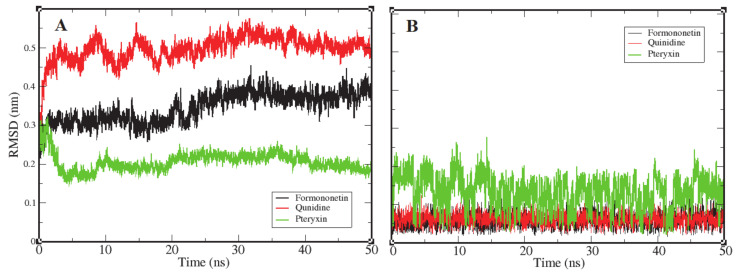
Representation of an RMSD plot of each complex in terms of their relationship between RMSD values and simulation time, shown in three different color codes. Panel (**A**) shows an RMSD plot of the backbone atoms of FimH protein receptor interacting with three ligand candidates, while panel (**B**) represents the RMSD plots of ligand candidates during simulation, which are color coded black (Formononetin), red (Quinidine), and green (Pteryxin). FimH protein complexes with Formononetin and Quinidine possessed high stability during simulation, while the third complex with Pteryxin demonstrated comparatively less stability.

Root mean square deviation (RMSD) is the most significant dynamic to explore in terms of conformational changes by means of stability in the structure and dynamic behavior of the receptor [69]. The complexes of formononetin, quinidine, and (+)-pteryxin with FimH obtained in molecular docking that interacted the best were further probed by MD simulation. The binding of ligands to their receptor protein can result in large conformational deviations in the resulting complex, specifically at the binding site. In this study, values of RMSD were increased at the beginning, with respect to the native structure of the RMSD. Figure 6A reports the RMSD of the backbone atoms of FimH protein as they interacted with each ligand molecule, where formononetin–FimH, quinidine–FimH and (+)-pteryxin–FimH interactions are represented with a black, red, and green color code, respectively. RMSD changes in all systems were initiated at the same point (~0.125 nm); however, in the formononetin–FimH complex, the conformational changes took place throughout the stabilization process. The change in the RMSD values in the formononetin–FimH complex indicated a small fluctuation until ~30 ns, and, afterwards, RMSD was found to be stable, remaining at around 0.35 nm in value. Due to the initial ups and downs in the RMSD values of the formononetin–FimH complex, the topologies of the structures were observed to significantly change during simulation. The observed initial RMSD change predicted the large conformational changes in regions near the binding pocket. Although the simulation lasted for 50 ns, the discrepancy from the initial structure within the first 30 ns was sufficient to point out the protein structures that were substantially denatured at binding sites and adjacent areas. While the RMSD values of the quinidine–FimH complex showed little turbulence until ~20 ns, the values reached a plateau later on. The value observed was greater than 0.3 nm initially; however, after 35 ns it went steady at 0.37 nm. In general, overall RMSD values fluctuated within 0.5 nm ± 0.05 nm throughout the simulation.

Like the above complexes, RMSD values showed frequent conformational changes and binding residue incorporation during the simulation analysis of the pteryxin–FimH complex. Conformational changes were observed at a functional domain near the binding site of the complex, and a considerable movement of the pteryxin ligand was also predicted at that site. Moreover, the RMSDs of all three ligand candidates were calculated to evaluate the behavior of the ligands within the binding site of the protein. Figure 6B shows the plot representation of RMSD for each ligand. Ligands (formononetin and quinidine) have generally shown high stability within the close contact residues of protein, whereas, in the case of pteryxin, this ligand demonstrated several fluctuations within the binding site. Due to the movement of the pteryxin ligand, an initial sudden elevation (~0.13 nm) followed by fluctuations until 0.15 ns was observed during the simulation. Although the RMSDs maintained a plateau later on, an average value of 0.21 nm was calculated throughout the simulation. Moreover, the difference in RMSDs due to the movement of pteryxin from the initial to the final frame at the binding site was observed as 0.15 nm, which revealed that pteryxin shows considerably weaker binding with interacting amino acid residues during the simulation. On the other hand, it was observed that formononetin and quinidine stably interacted during simulation, and the pattern of contact between the binding residues and both ligands (formononetin and quinidine) predicted regulation of the activity of the FimH protein.

Consequently, the RMSDs of the formononetin–FimH and quinidine–FimH complexes were found to have comparatively more stable trajectories than the (+)–pteryxin–FimH complex during simulation analysis (Figure 6).

### 3.3. Principle Component Analysis

**Figure 7:**
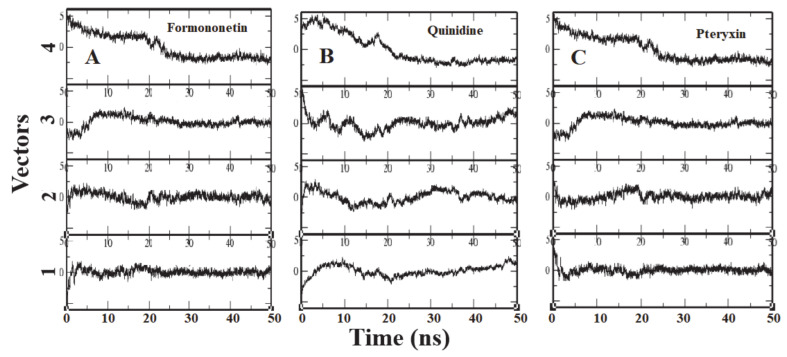
Each panel (**A**–**C**) reports the calculation of projection of particular trajectory on eigenvectors for each three complexes. The RMSDs for every four eigenvectors are depicted as four subplots in each panel. Plot shows the projections of each vector are fitted to the structure in the eigenvector.

To identify the overall patterns of motion in the complexes during simulation, we utilized principal components analysis (PCA) for the three complexes. The covariance matrices of the simulations were calculated to produce the eigenvectors, and then a screening of the trajectories was performed around each of the diverse eigenvectors. Furthermore, the dominant motions during a four-vector RMSD for each complex were observed (Figure 7). A large portion of the overall variations in the receptor protein can be explained by a few reduced amplitude eigenvectors with large eigenvalues. The analysis illustrated that the fourth eigenvector accounts for the depletion of the RMSD value, although the rest of the three vectors showed elevation at the initial time of the simulation. It was shown that after ~8 ns the RMSD values reached a plateau and maintained their value until the end of the simulation (Figure 7).

**Figure 8:**
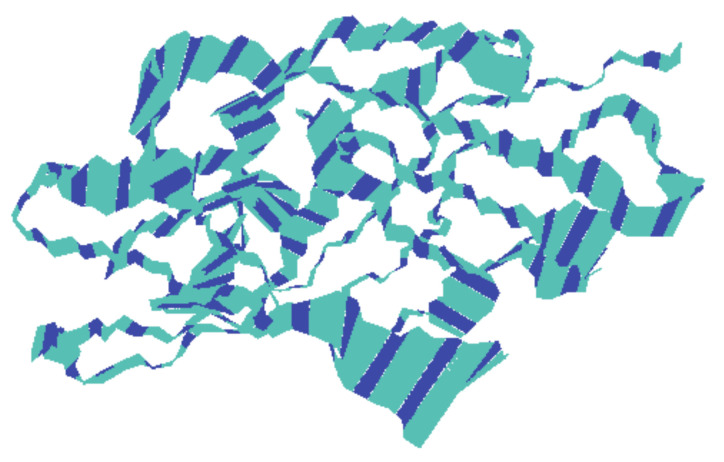
The motion of the backbone atoms of the protein receptor during simulation of the trajectory as calculated by principal component analysis. The width of the bands is proportional to amplitude.

To know the movement of the backbone atoms of the receptor protein, 100 frames of the first principal component were collectively loaded into VMD, which show the motility of the residues (Figure 8), although there might be differences between each complex with respect to the motions sampled. The width of the band is proportional to amplitude; a narrow band signifies those segments that hardly moved, while wide bands signify the sections most affected by the transitions. The middle domain of the protein showed less movement than the adjacent domains that predicted the stability of the middle domain. The rest of the protein exhibited comparatively wider bands, which might be due to an increase in functionality.

It is worth mentioning that the binding energy values and MD simulations obtained can only demonstrate the binding efficacy and stability of inhibitors with the target protein. Further benchwork experiments are required to optimize these compounds (formononetin, (+)-pteryxin, and quinidine) as promising SGLT2 inhibitors with add-on FimH inhibition potential that might reduce the probability of uropathogenic side effects.

## 4. Conclusions

SGLT2 inhibitors are a newer class of drugs that have enormous anti-diabetic potential. Unfortunately, the side effects, specifically those that contribute to the development of UTIs, have impeded their success rate. Several clinicians, as well as diabetic patients, are still in a dilemma over the use of this class of anti-diabetic medication. In contrast, FimH is blooming as a potential alternative target for UTI treatment, and FimH inhibitors are currently under development. These inhibitors act against the uropathogenic bacterial strains adherence to the mucosal surface of the urinary tract, resulting in a lower chance of encountering resistance. In the present study, we docked natural SGLT2 inhibitors with FimH target proteins to predict their FimH interaction potential. To the best of our knowledge, this is the first time that SGLT2 inhibitors have been explored in terms of their use against FimH. Our findings suggest that among all SGLT2 inhibitors examined, formononetin, (+)-pteryxin, and quinidine exhibited strong interactions with the FimH protein with high binding energy, in comparison to the positive control heptyl α-d-mannopyranoside. On the other hand, the FDA-approved SGLT2 inhibitor canagliflozin indicated lower interaction with FimH. Thus, we hypothesize that if explored further, natural SGLT2 inhibitors might reduce the probability of UTIs when used against FimH. The authors anticipate researchers to duly use the findings of this study to design more versatile and potent dual inhibitors against SGLT2 and FimH to cope with the uropathogenic side effects of SGLT2 inhibitor class anti-diabetic medications. 

## Figures and Tables

**Table 1:** Physicochemical properties of natural sodium–glucose co-transporter 2 (SGLT2) inhibitors and control compounds.

S.No.	Compound Name	Physiochemical Parameters							
		% of Absorption **	Topological Polar Surface Area (Å)^2^	Molecular Weight	cLogP ***	Hydrogen Bond Donors	Hydrogen Bond Acceptors	Number of Rotatable Bonds	Lipinski’s Violation
	Rule	-	-	<500	≤5	<5	<10	≤10	≤1
**1.**	Acerogenin B	91.85	49.69	298.38	4.50	2	3	0	0
**2.**	Formononetin	89.76	55.76	268.26	2.24	1	4	2	0
**3.**	(−)-kurarinone	75.80	96.22	438.51	6.11	3	6	7	1
**4.**	(+)-pteryxin	78.59	88.13	386.39	3.34	0	7	5	0
**5.**	Quinidine	93.27	45.59	324.42	2.61	1	4	4	0
**6.**	Canagliflozin *	68.15	118.39	444.52	3.27	4	5	5	0
**7.**	Heptyl α-d-mannopyranoside *	74.71	99.38	278.34	0.485	4	6	8	0

* Control drugs/compounds; ** Percentage of Absorption (% of Absorption) was calculated by the following equation: % of Absorption = 109 − (0.345 × Topological Polar Surface Area); *** Logarithm of the compound partition coefficient between n-octanol and water.

**Table 2:** Toxicity potential of natural SGLT2 inhibitors and control compounds.

S.No.	Compound Name	Toxicity Risks			
		Mutagenic	Tumorigenic	Reproductive Effect	Irritant
**1.**	Acerogenin B	None	None	None	None
**2.**	Formononetin	None	None	None	None
**3.**	(−)-kurarinone	None	None	None	None
**4.**	(+)-pteryxin	None	None	None	High
**5.**	Quinidine	None	None	None	None
**6.**	Canagliflozin *	None	None	None	None
**7.**	Heptyl α-d-mannopyranoside *	None	None	None	None

* Control drugs/compounds.

**Table 3:** The docking results of the molecular interactions of SGLT2 and FimH with natural SGLT2 inhibitors and control compounds.

Compounds	SGLT2		FimH	
	Binding Energy (kcal/mol)	Inhibition Constant (µM)	Binding Energy (kcal/mol)	Inhibition Constant (µM)
Canagliflozin *	−7.23	5.04	−3.56	2450
Acerogenin B	−6.30	24.25	−4.40	598.52
Formononetin	−7.63	2.57	−5.65	71.95
(−)-kurarinone	−7.23	5.03	−3.93	1310
(+)-pteryxin	−9.01	0.248	−5.50	92.97
Quinidine	−8.77	0.371	−5.70	66.40
Heptyl α-d-mannopyranoside **	-	-	−4.46	109.49

* Control compound for SGLT2; ** Control compound for FimH.

## Data Availability

Not applicable.

## References

[1] Leung M.Y., Pollack L.M., Colditz G.A., Chang S.H. (2015). Life years lost and lifetime health care expenditures associated with diabetes in the U.S., national health interview survey, 1997–2000. Diabetes Care.

[2] Shah B.R., Hux J.E. (2003). Quantifying the risk of infectious diseases for people with diabetes. Diabetes Care.

[3] Thomsen R.W., Hundborg H.H., Lervang H.H., Johnsen S.P., Schonheyder H.C., Sorensen H.T. (2005). Diabetes mellitus as a risk and prognostic factor for community-acquired bacteremia due to enterobacteria: A 10-year, population-based study among adults. Clin. Infect. Dis..

[4] Thomsen R.W., Hundborg H.H., Lervang H.H., Johnsen S.P., Sorensen H.T., Schonheyder H.C. (2004). Diabetes and outcome of community-acquired pneumococcal bacteremia: A 10-year population based cohort study. Diabetes Care.

[5] Dardi I., Kouvatsos T., Jabbour S.A. (2016). SGLT2 inhibitors. Biochem. Pharmacol..

[6] Benfield T., Jensen J.S., Nordestgaard B.G. (2007). Influence of diabetes and hyperglycaemia on infectious disease hospitalization and outcome. Diabetologia.

[7] Boyko E.J., Fihn S.D., Scholes D., Abraham L., Monsey B. (2005). Risk of urinary tract infection and asymptomatic bacteriuria among diabetic and nondiabetic postmenopausal women. Am. J. Epidemiol..

[8] Geerlings S., Fonseca V., Castro-Diaz D., List J., Parikh S. (2014). Genital and urinary tract infections in diabetes: Impact of pharmacologically induced glucosuria. Diabetes Res. Clin. Pract..

[9] U.S. Food and Drug Administration (2015). FDA Drug Safety Communication: FDA Revises Labels of SGLT2 Inhibitors for Diabetes to Include Warnings About Too Much Acid in the Blood and Serious Urinary Tract Infections. http://www.fda.gov/Drugs/DrugSafety/ucm475463.htm.

[10] Zinman B., Wanner C., Lachin J.M., Fitchett D., Bluhmki E., Hantel S., Mattheus M., Devins T., Johansen O.E., Woerle H.J. (2015). Empagliflozin, cardiovascular outcomes, and mortality in type 2 diabetes. N. Engl. J. Med..

[11] Neal B., Perkovic V., Mahaffey K.W., de Zeeuw D., Fulcher G., Erondu N., Shaw W., Law G., Desai M., Matthews D.R. (2017). Canagliflozin and cardiovascular and renal events in type 2 diabetes. N. Engl. J. Med..

[12] Fronzes R., Remaut H., Waksman G. (2008). Architectures and biogenesis of non-flagellar protein appendages in Gram-negative bacteria. EMBO J..

[13] Mydock-McGrane L.K., Cusumano Z.T., Janetka J.W. (2016). Mannose-derived FimH antagonists: A promising anti-virulence therapeutic strategy for urinary tract infections and Crohn’s disease. Expert Opin. Ther. Pat..

[14] Shimokawa Y., Akao Y., Hirasawa Y., Awang K., Hadi A.H., Sato S., Aoyama C., Takeo J., Shiro M., Morita H. (2010). Gneyulins A and B, stilbene trimers, and noidesols A and B, dihydroflavonol-C-glucosides, from the bark of Gnetum gnemonoides. J. Nat. Prod..

[15] Sato S., Takeo J., Aoyama C., Kawahara H. (2007). Na+-glucose cotransporter (SGLT) inhibitory flavonoids from the roots of Sophora flavescens. Bioorg. Med. Chem..

[16] Oranje P., Gouka R., Burggraaff L., Vermeer M., Chalet C., Duchateau G., van der Pijl P., Geldof M., de Roo N., Clauwaert F. (2019). Novel natural and synthetic inhibitors of solute carriers SGLT1 and SGLT2. Pharmacol. Res. Perspect..

[17] Abgottspon D., Rölli G., Hosch L., Steinhuber A., Jiang X., Schwardt O., Cutting B., Smiesko M., Jenal U., Ernst B. (2010). Development of an Aggregation Assay to Screen FimH Antagonists. J. Microbiol. Methods.

[18] Lipinski C.A., Lombardo F., Dominy B.W., Feeney P.J. (2001). Experimental and computational approaches to estimate solubility and permeability in drug discovery and development settings. Adv. Drug Deliv. Rev..

[19] Zhao Y.H., Abraham M.H., Le J., Hersey A., Luscombe C.N., Beck G., Sherborne B., Cooper I. (2002). Rate-limited steps of human oral absorption and QSAR studies. Pharm. Res..

[20] Rizvi S.M.D., Shakil S., Haneef M.A. (2013). simple click by click protocol to perform docking: AutoDock 4.2 made easy for non-bioinformaticians. EXCLI J..

[21] Spoel D.V.D., Lindahl E., Hess B., Groenhof G., Mark A.E., Berendsen H.J. (2005). GROMACS: Fast, flexible, and free. J. Comput. Chem..

[22] Pronk S., Páll S., Schulz R., Larsson P., Bjelkmar P., Apostolov R., Shirts M.R., Smith J.C., Kasson P.M., van der Spoel D. (2013). GROMACS 4.5: A high-throughput and highly parallel open source molecular simulation toolkit. Bioinformatics.

[23] Oostenbrink C., Villa A., Mark A.E., van Gunsteren W.F. (2004). A biomolecular force field based on the free enthalpy of hydration and solvation: The GROMOS force-field parameter sets 53A5 and 53A6. J. Comput. Chem..

[24] Toukan K., Rahman A. (1985). Molecular-dynamics study of atomic motions in water. Phys. Rev. B.

[25] Essmann U., Perera L., Berkowitz M.L. (1995). A smooth particle mesh Ewald method. J. Chem. Phys..

[26] Hess B., Bekker H., Berendsen H.J.C., Fraaije J.G.E.M. (1997). LINCS: A linear constraint solver for molecular simulations. J. Comput. Chem..

[27] Schüttelkopf A.W., Van Aalten D.M. (2004). PRODRG: A tool for high-throughput crystallography of protein-ligand complexes. Acta Crystallogr. Sect. D Biol. Crystallogr..

[28] Seo E., Lee E.K., Lee C.S., Chun K.H., Lee M.Y., Jun H.S. (2014). Psoraleacorylifolia L. seed extract ameliorates streptozotocin-induced diabetes in mice by inhibition of oxidative stress. Oxid. Med. Cell Longev..

[29] Monteiro M., Farah A., Perrone D., Trugo L.C., Donangelo C. (2007). Chlorogenic acid compounds from coffee are differentially absorbed and metabolized in humans. J. Nutr..

[30] Mbaze L.M., Poumale H.M., Wansi J.D. (2007). alpha-Glucosidase inhibitory pentacyclic triterpenes from the stem bark of Fagara tessmannii (Rutaceae). Phytochemistry.

[31] Abdel-Zaher A.O., Salim S.Y., Assaf M.H., Abdel-Hady R.H. (2005). Antidiabetic activity and toxicity of Zizyphus spina-christi leaves. J. Ethnopharmacol..

[32] Meng X.Y., Zhang H.X., Mezei M., Cui M. (2011). Molecular docking: A powerful approach for structure-based drug discovery. Curr. Comput. Aided Drug Des..

[33] Udrescu L., Bogdan P., Chiş A., Sîrbu I.O., Topîrceanu A., Văruţ R.M., Udrescu M. (2020). Uncovering New Drug Properties in Target-Based Drug-Drug Similarity Networks. Pharmaceutics.

[34] Pinzi L., Rastelli G. (2019). Molecular Docking: Shifting Paradigms in Drug Discovery. Int. J. Mol. Sci..

[35] Rajesh R., Naren P., Sudha V., Unnikrishnan M.K., Pandey S., Varghese M., Gang S. (2010). Sodium Glucose Co transporter 2 (SGLT2) Inhibitors: A New Sword for the Treatment of Type 2 Diabetes Mellitus. Int. J. Pharma Sci. Res..

[36] Shaikh S., Rizvi S.M.D., Shakil S., Riyaz S., Biswas D., Jahan R. (2016). Forxiga (Dapagliflozin): Plausible role in the treatment of diabetes associated neurological disorders. Biotechnol. Appl. Biochem..

[37] Rizvi S.M.D., Shakil S., Biswas D., Shakil S., Shaikh S., Bagga P., Kamal M.A. (2014). Invokana (Canagliflozin) as a Dual Inhibitor of Acetylcholinesterase and Sodium Glucose Co-Transporter 2: Advancement in Alzheimer’s Disease-Diabetes Type 2 Linkage via an Enzoinformatics Study. CNS Neurol. Disord.-Drug Targets.

[38] Kaushal S., Singh H., Thangaraju P., Singh J. (2014). Canagliflozin: A novel SGLT2 inhibitor for Type 2 Diabetes Mellitus. N. Am. J. Med. Sci..

[39] Díez-Sampedro A., Barcelona S. (2011). Sugar binding residue affects apparent Na affinity and transport stoichiometry in mouse sodium/glucose cotransporter type 3B. J. Biol. Chem..

[40] Liu T., Krofchick D., Silverman M. (2009). Effects on conformational states of the rabbit sodium/glucose cotransporter through modulation of polarity and charge at glutamine 457. Biophys. J..

[41] Wright E.M., Turk E., Martin M.G. (2002). Molecular basis for glucose galactose malabsorption. Cell Biochem. Biophys..

[42] Choi C.I. (2016). Sodium-Glucose Cotransporter 2 (SGLT2) Inhibitors from Natural Products: Discovery of Next-Generation Antihyperglycemic Agents. Molecules.

[43] Ito S., Hosaka T., Yano W., Itou T., Yasumura M., Shimizu Y., Kondo T. (2018). Metabolic effects of tofogliflozin are efficiently enhanced with appropriate dietary carbohydrate ratio and are distinct from carbohydrate restriction. Physiol. Rep..

[44] Okauchi S., Shimoda M., Obata A., Kimura T., Hirukawa H., Kohara K., Kaneto H. (2016). Protective effects of SGLT2 inhibitor Luseogliflozin on pancreatic β-cells in obese type 2 diabetic db/db mice. Biochem. Biophys. Res. Commun..

[45] Arai H., Hirasawa Y., Rahman A., Kusumawati I., Zaini N.C., Sato S., Aoyama C., Takeo J., Morita H. (2010). Alstiphyllanines E-H, picraline and ajmaline-type alkaloids from *Alstonia macrophylla* inhibiting sodium glucose cotransporter. Bioorg. Med. Chem..

[46] Qu Y., Chan J.Y., Wong C.W., Cheng L., Xu C., Leung A.W., Lau C.B. (2015). Antidiabetic Effect of Schisandrae Chinensis Fructus Involves Inhibition of the Sodium Glucose Cotransporter. Drug Dev. Res..

[47] Cao X., Zhang W., Yan X., Huang Z., Zhang Z., Wang P., Shen J. (2016). Modification on the O-glucoside of Sergliflozin-A: A new strategy for SGLT2 inhibitor design. Bioorg. Med. Chem. Lett..

[48] Wilding J.P., Charpentier G., Hollander P., González-Gálvez G., Mathieu C., Vercruysse F., Usiskin K., Law G., Black S., Canovatchel W. (2013). Efficacy and safety of canagliflozin in patients with type 2 diabetes mellitus inadequately controlled with metformin and sulphonylurea: A randomized trial. Int. J. Clin. Pract..

[49] Rosenstock J., Jelaska A., Frappin G., Salsali A., Kim G., Woerle H.J., Broedl U.C., EMPA-REG MDI Trial Investigators (2014). Improved glucose control with weight loss, lower insulin doses, and no increased hypoglycemia with empagliflozin added to titrated multiple daily injections of insulin in obese inadequately controlled type 2 diabetes. Diabetes Care.

[50] Jabbour S.A., Hardy E., Sugg J., Parikh S. (2014). Dapagliflozin is effective as add-on therapy to sitagliptin with or without metformin: A 24-Week, multicenter, randomized, double blind, placebo-controlled study. Diabetes Care.

[51] Bode B., Stenlof K., Harris S., Sullivan D., Fung A., Usiskin K., Meininger G. (2015). Long-term efficacy and safety of canagliflozin over 104 weeks in patients aged 55–80 years with type 2 diabetes. Diabetes Obes. Metab..

[52] Haering H.U., Merker L., Christiansen A.V., Roux F., Salsali A., Kim G., Meinicke T., Woerle H.J., Broedl U.C., EMPA-REG EXTEND™ METSU Investigators (2015). Empagliflozin as add-on to metformin plus sulphonylurea in patients with type 2 diabetes. Diabetes Res. Clin. Pract..

[53] Ronald A.R., Nicolle L.E., Stamm E., Krieger J., Warren J., Schaeffer A., Naber K.G., Hooton T.M., Johnson J., Chambers S. (2001). Urinary tract infection in adults: Research priorities and strategies. Int. J. Antimicrob. Agents.

[54] Anderson G.G., Palermo J.J., Schilling J.D., Roth R., Heuser J., Hultgren S.J. (2003). Intracellular bacterial biofilm-like pods in urinary tract infections. Science.

[55] Mydock-McGrane L.K., Hannan T.J., Janetka J.W. (2017). Rational Design Strategies for FimH Antagonists: New Drugs on the Horizon for Urinary Tract Infection and Crohn’s Disease. Expert Opin. Drug Discov..

[56] Wellens A., Garofalo C., Nguyen H., Van Gerven N., Slättegård R., Hernalsteens J.P., Wyns L., Oscarson S., De Greve H., Hultgren S. (2008). Intervening with urinary tract infections using anti-adhesives based on the crystal structure of the FimH-oligomannose-3 complex. PLoS ONE.

[57] Hung C.S., Bouckaert J., Hung D., Pinkner J., Widberg C., DeFusco A., Auguste C.G., Strouse R., Langermann S., Waksman G. (2002). Structural basis of tropism of *Escherichia coli* to the bladder during urinary tract infection. J. Mol. Microbiol..

[58] Chen S.L., Hung C.S., Pinkner J.S., Walker J.N., Cusumano C.K., Li Z., Bouckaert J., Gordon J.I., Hultgren S. (2009). Positive selection identifies an *in vivo* role for FimH during urinary tract infection in addition to mannose binding. Proc. Natl. Acad. Sci. USA.

[59] Mousavifar L., Vergoten G., Charron G., Roy R. (2019). Comparative study of aryl O-, C-, and S-mannopyranosides as potential adhesion inhibitors toward uropathogenic *E. coli* FimH. Molecules.

[60] Mousavifar L., Touaibia M., Roy R. (2018). Development of mannopyranoside therapeutics against adherent-invasive *Escherichia coli* infections. Acc. Chem. Res..

[61] Sarshar M., Behzadi P., Ambrosi C., Zagaglia C., Palamara A.T., Scribano D. (2020). FimH and anti-adhesive therapeutics: A disarming strategy against uropathogens. Antibiotics.

[62] Ribić R., Meštrović T., Neuberg M., Kozina G. (2018). Effective anti-adhesives of uropathogenic *Escherichia coli*. Acta Pharm..

[63] Rafsanjany N., Senker J., Brandt S., Dobrindt U., Hensel A. (2015). In vivo consumption of cranberry exerts ex vivo antiadhesive activity against FimH-Dominated uropathogenic *Escherichia coli*: A combined in vivo, ex vivo, and in vitro study of an extract from vaccinium macrocarpon. J. Agric. Food Chem..

[64] Ansari M.A., Shaikh S., Shakil S., Rizvi S.M. (2014). An enzoinformatics study for prediction of efficacies of three novel penem antibiotics against New Delhi metallo-β-lactamase-1 bacterial enzyme. Interdiscip. Sci. Comput. Life Sci..

[65] Chaturvedi N., Yadav B.S., Pandey P.N., Tripathi V. (2017). The effect of β-glucan and its potential analog on the structure of Dectin-1 receptor. J. Mol. Graph. Model.

[66] Rizvi S.M.D., Shakil S., Zeeshan M., Khan M.S., Shaikh S., Biswas D., Ahmad A., Kamal M.A. (2014). An enzoinformatics study targeting polo-like kinases-1 enzyme: Comparative assessment of anticancer potential of compounds isolated from leaves of Ageratum houstonianum. Pharmacogn. Mag..

[67] Verma A., Rizvi S.M.D., Shaikh S., Ansari M.A., Shakil S., Ghazal F., Siddiqui M.H., Haneef M., Rehman A. (2014). Compounds isolated from Ageratum houstonianum inhibit the activity of matrix metalloproteinases (MMP-2 and MMP-9): An oncoinformatics study. Pharmacogn. Mag..

[68] Erondu N., Desai M., Ways K., Meininger G. (2015). Diabetic ketoacidosis and related events in the canagliflozin type 2 diabetes clinical program. Diabetes Care.

[69] Kuzmanic A., Zagrovic B. (2010). Determination of ensemble-average pairwise root mean-square deviation from experimental B-factors. Biophys. J..

